# Nutrient intakes and metabolomic profiles associated with animal source energy percentage in children’s diets

**DOI:** 10.1038/s41598-025-02114-8

**Published:** 2025-05-27

**Authors:** Topi Hovinen, Elina Kettunen, Maijaliisa Erkkola, Anu Suomalainen, Riitta Freese, Liisa Korkalo

**Affiliations:** 1 Research Programs Unit, Stem Cells and Metabolism, University of Helsinki, 00290 Helsinki, Finland; 2 Department of Food and Nutrition, University of Helsinki, 00014 Helsinki, Finland; 3 HUS Diagnostic Center, Helsinki University Hospital, 00290 Helsinki, Finland; 4 HiLife, University of Helsinki, 00014 Helsinki, Finland

**Keywords:** Vegan diet, Plant-based dietary pattern, Diet classification, ASEP, Indoleacrylic acid, Cholesterol, Biomarkers, Metabolism

## Abstract

The purpose of the study was to calculate the relative intake of animal source energy (animal source energy percentage, ASEP) and examine its utility in nutrition and metabolic research with a special attention on children. In addition, the capacity of ASEP to identify potential metabolic biomarkers of plant-forward diets was tested. Four-day food records and 7-day dietary screeners of 51 Finnish children aged 1–7 years, consuming a vegan (*n* = 7), pesco-/lacto-/ovo-vegetarian (*n* = 11) or omnivorous diet (*n* = 33), were used for assessing general performance of ASEP as diet measure. Blood samples of 40 children (six vegans, eight vegetarians, 26 omnivores) were analyzed for plasma LDL cholesterol, erythrocyte folate concentration and serum untargeted MS-metabolomics. Estimated ASEPs varied between 0% and 52%. Vegetarians and omnivores showed overlapping ASEPs. ASEP had a strong positive correlation with saturated fatty acid (percent of energy, E%; *r* = 0.785) and cholesterol intake (*r* = 0.715) and a strong negative correlation with fibre (*r* = − 0.811) and polyunsaturated fatty acid (E%; *r* = − 0.819) intake. ASEP also correlated with plasma LDL cholesterol (*r* = 0.699) concentration. Serum indoleacrylic acid (IAA) is suggested to be further investigated as a possible biomarker of plant-forward diet. ASEP provides a useful tool for objective and quantitative assessment of diet characteristics in research.

**Supplementary Information:**

The online version contains supplementary material available at 10.1038/s41598-025-02114-8.

## Introduction

Consumption of animal source foods (ASF), including meat, fish, seafood, eggs, and dairy, has impacts on both human and environmental health. Subgroups of ASF represent concentrated sources of many nutrients, including essential amino- and fatty acids, iron, calcium, and vitamins A, D and B12^1^. At the same time, high intake of red and processed meat is linked with chronic diseases^2^ and in high-income countries, substitution of animal protein with plant protein is likely to confer health benefits^3^. Growing concern over environmental issues, such as climate change and biodiversity loss, has encouraged integration of sustainability aspects into the dietary guidelines^4^, with a major focus on a dietary shift towards increasingly plant-based diets^5^.

In research on plant-based diets, diet groups, such as vegan or vegetarian, are typically compared against the omnivorous diet. Despite the increased media attention around veganism and the growing market of plant-based food products, the proportion of vegans – those adhering to an exclusively plant-based diet – remains low in Western societies^6^. Even though the term vegetarian is often taken to refer to a diet that is more plant-based than an omnivorous diet, these terms fail to describe quantitative differences in total ASF consumption. For the general population, a gradual transition towards more plant-based diet is likely to be more feasible than complete exclusion of certain ASF^7^. At the same time, the evolving terminology in nutrition science, where dietary patterns are described as “plant-predominant”, “plant-forward” or “flexitarian”^8^, also suggests a need for methods to assess such dietary patterns.

Accurate and objective ways to describe dietary patterns are crucial for assessing environmental impacts, but also for identifying risks and benefits within the proposed shift towards plant-forward diets. The metabolic and health consequences of such dietary shift in children deserve special attention, as due to rapid growth and development, children are more susceptible to nutrient deficiencies than adults^9^. At the same time, dietary patterns established in childhood may modify the future risk of chronic diseases^10,11^. For instance, childhood LDL-cholesterol (LDL-C) concentration, a risk factor with strong evidence of lifelong tracking^12^, builds up the cumulative risk burden of cardiovascular disease over the life course.

Perignon et al.^13^ examined the animal-to-plant ratio of a diet and its relationship with nutrient bioavailability. Looking at the consumption of ASF within 7-d food records, they calculated the share of both animal protein and animal energy intake. We developed this approach further to characterize diets based on the percentage of energy intake from ASF. Here, as an example case of use of this method, we describe the calculation of animal source energy intake percentage (ASEP) from the food records of a sample of Finnish daycare children aged 1–7 years, and its value when assessing nutrient intakes and statuses, with a special focus on nutrients relevant for cardiovascular health. We evaluate the clinical and public health-related relevance of ASEP through its correlations with established clinical blood biomarker levels and by applying ASEP to the EAT-Lancet commission’s planetary health reference diet^5^. Finally, we combine ASEP with untargeted metabolomics to explore candidate serum biomarkers of animal or plant source food use.

## Results

### Participant characteristics

Table 1 summarizes the characteristics of the study participants and their dietary intakes. A blood sample was not obtained from eleven participants and these participants were excluded from correlation analyses with biological measurements. All participants were apparently healthy.

**Table 1:** The characteristics of the study participants and their calculated dietary intakes.

n	51
Participant characteristics	
Sex, F:M (n:n)	23:28
Age, years	3.49 (1.43–7.10)
BMI SD score	0.30 (-2.69–3.06)
Dietary intakes	
Energy, kJ/d	5244 (3386–8115)
Energy, kcal/d	1253 (809–1940)
Fat, E%	33.0 (19.3–41.8)
SAFA, E%	10.7 (4.9–17.9)
MUFA, E%	11.3 (6.4–15.2)
PUFA, E%	6.2 (3.0–12.4)
Cholesterol, mg/MJ	18.6 (0.1–51.9)
Cholesterol, mg/d	100.4 (0.5–249.5)
Protein, E%	15.5 (10.1–21.3)
Carbohydrates, E%	47.9 (41.3–65.0)
Fibre, g/MJ	3.5 (1.7–6.2)
Fibre, g/d	17.3 (7.1–38.4)
Folate, µg/d	175 (94–505)

The values are median (minimum - maximum) unless otherwise indicated. ‘F’ for female and ‘M’ for male. Age of the child at the time of the first food record day. ‘E%’ represents the percentage of total energy. BMI standard deviation (SD) score is calculated from the Finnish population growth data^14^.

### Food item scoring

Of the 719 food items listed in the food records, a majority (*n* = 488, 68%) was estimated to originate exclusively from plant-sources. Correspondingly, 168 (23%) food items were classified as completely animal-origin. The remaining 63 (9%) food items contained both plant- and animal-origin ingredients. Most of these (21 food items, together accounting for 46% of energy derived from mixed-origin foods) were spreadable fats. Collectively the mixed-origin food items accounted for 0.0%–20.3% (median 4.7%) of the estimated total energy intake among the 43 non-vegan participants of our sample.

### Animal source energy percentage

**Fig. 1:**
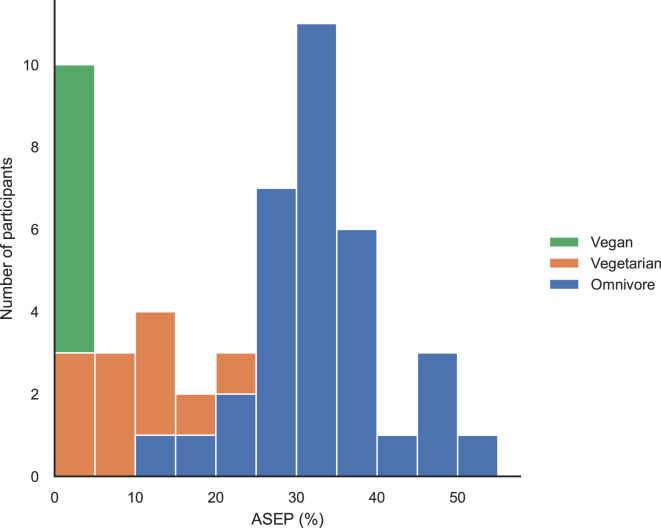
The distribution of animal source energy percentages (ASEPs) calculated from the 3- or 4-day food records (*n* = 51) across the diet categories.

The distribution of ASEPs among the children from whom 3- or 4 day-food record data was available (*n* = 51) is presented in Fig. 1. Among the study participants, ASEP varied between 0% and 52% (median 27%). All children categorized as vegans obtained an ASEP value of 0%. For vegetarians, ASEP ranged from 0% to 20% (median 8%), with three vegetarians obtaining an ASEP below 5%. Among the participants classified as omnivores, ASEP varied from 11% to 52% (median 33%).

Within the EAT-Lancet commissions’ planetary health diet^5^, 340 kcal of total daily energy intake of 2500 kcal originated from animal source food groups, resulting in ASEP estimate of 14%.

### Variability of ASEP

The estimated intraindividual standard deviation of daily ASEP, $\:\widehat{{s}_{ASEP}}$, based on the vegetarian and omnivore food records was 0.0813 (≈ 8% points). Distribution of the individual $\:\widehat{{s}_{ASEP,i}}$ is shown in Supplementary Fig. 1. Table 2 shows the required days of food record for selected maximum absolute deviation limits *a* of an individual.

**Table 2:** Required days of food record to achieve maximum absolute deviation *a* (or *a* * 100%) for individual animal source energy percentage (ASEP) with a confidence level of 95%. Calculation method is described in detail in the “Methods” Section.

	Required days of food record
a = 0.005	1015
a = 0.01	254
a = 0.02	64
a = 0.05	11
a = 0.1	3

The estimated intraindividual correlation coefficient r_i_ from all possible 246 random pairs of daily ASEPs in our data was 0.537. This yields four required days of food record to ensure error term greater than 0.9 for correlation analyses.

### Associations between ASEP, nutrient intakes and clinical biomarkers

**Fig. 2:**
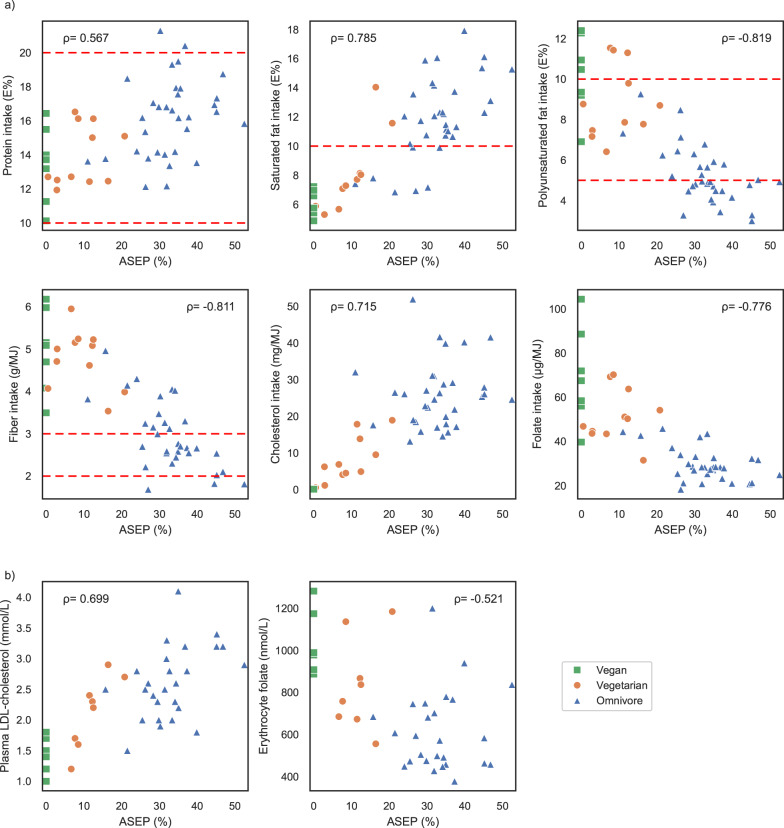
In (**a**), scatter plots visualize the correlations between animal source energy percentage (ASEP) and dietary intake of protein (as percentage of energy [E%]), saturated fatty acids (SAFA, E%), polyunsaturated fatty acids (PUFA, E%), fibre (g/MJ), cholesterol (mg/MJ), and folate (µg/MJ), (*n* = 51). The dashed red lines represent the Finnish dietary recommendations for children over two years of age for protein (10–20 E%), SAFA (< 10E%), PUFA (5–10 E%), and fiber (2–3 g/MJ)^25^. In (**b**), scatter plots show correlations of ASEP and plasma LDL cholesterol (mmol/L) and erythrocyte folate concentration (nmol/L), (*n* = 40). Spearman’s rho was calculated using Spearman’s rank correlation coefficient.

Spearman correlation analyses between ASEP and several nutrient intakes, plasma LDL-C and erythrocyte folate levels are presented in Fig. 2. Of macronutrients, ASEP did not correlate with fat or carbohydrate intake, but had a positive correlation with protein (percent of energy, E%) intake (*r* = 0.567). ASEP had a strong positive correlation with saturated fatty acid (SAFA, E%; *r* = 0.785) and high negative correlations with polyunsaturated fatty acid (PUFA, E%; *r* = − 0.819) and fibre (g/MJ; *r* = − 0.811) intakes. In addition, ASEP correlated strongly with cholesterol intake (mg/MJ; *r* = 0.715) and negatively with folate intake (µg/MJ, *r* = − 0.776). Of blood biomarkers, a positive correlation of ASEP with plasma LDL-C (*r* = 0.699) and a negative correlation with erythrocyte folate (*r* = − 0.521) levels was detected. In our sample, the LDL-C concentrations varied between 1.0 and 4.1 mmol/L. Moreover, six (15%) of the participants fulfilled the criteria for hypercholesterolemia (LDL-C > 3.0 mmol/L). A complete list of the correlations of ASEP with the chosen 10 nutrient intakes and two clinical biomarkers of interest is presented in Supplementary Table 1.

### Serum biomarkers for plant-based diet

**Fig. 3:**
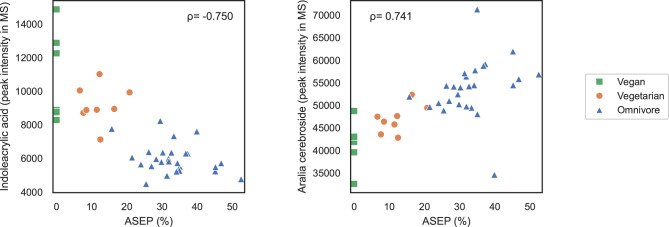
The association of animal source energy percentage (ASEP) with serum indoleacrylic acid and aralia cerebroside, detected by untargeted MS-metabolomics. Spearman’s rho included in the figure was calculated using Spearman’s rank correlation coefficient.

The 20 metabolites with the highest absolute Spearman rank correlation coefficients with ASEP are presented and their potential relevance is summarized in Supplementary Table 2. The most significant positive and negative correlations are illustrated as scatter plots in Fig. 3. The interrelations of these metabolites are demonstrated with a correlation-based clustering in Supplementary Fig. 2. Fifteen of these top metabolites have a positive correlation with ASEP, suggesting higher levels in individuals with a more animal-based diet, and five of the listed top metabolites show negative correlation with ASEP. The most significant negative correlation was with a known plant growth hormone indoleacrylic acid (IAA)^15^. Other metabolites with a negative correlation included lysophosphatidylcholine with palmitic acid as the fatty acid moiety (LysoPC(16:0)) and pipecolate. Aralia cerebroside was the metabolite with the highest positive correlation with ASEP. Out of the 15 metabolites positively correlating with ASEP, ten were glycerophospholipids.

## Discussion

Here, we suggest a novel approach for dietary pattern assessment. This method enables accurate, easy-to-interpret and quantitative investigation across distinct diet types, and allows use of statistical methods assuming continuous variables. We used ASEP to assess food intake in Finnish daycare children and found a remarkable correlation between ASEP and intakes of several nutrients. We also found that ASEP correlated with plasma LDL-C concentration in our sample with high variation in LDL-C concentrations. Finally, we combined ASEP to untargeted MS-metabolomics data of serum samples to reveal notable correlations with various metabolites, including indoleacrylic acid (IAA), an plant growth hormone that human cells are not able to synthesize^15^. We suggest further studies to examine the potential role of IAA as a serum biomarker of the proportion of plant-based energy in diet.

When examining distributions of ASEP ranges, we observed substantial overlap of ASEP between children with omnivorous and vegetarian diets. Here, we report an estimated median ASEP of 33% for daycare children with omnivorous diet in Finnish urban environment. The children with vegetarian diets, who due to the recruitment method were all consuming vegan diets at daycare, had an estimated median ASEP of 8%. Globally children’s ASF use has been increasing, as reported by Miller et al.^16^. They detected a rise in children’s and adolescents’ ASF consumption during 1990–2018 in all regions except sub-Saharan Africa. This study lacked, however, the information regarding the intake of poultry, a major global source of ASF. In many European countries, including Finland, over 35% of the food supply’s energy content is estimated to derive from animal products^17^. Among adults, a study on randomly selected French participants (*n* = 1899) reported a mean share of animal energy of 34.5% (SD +/- 7.4) when calculated from 7-d food records^13^. In accordance with our findings in children, the French study reported intake of animal source energy for women between 10.1% and 66.9%, with comparable values for men. The large within-diet group heterogeneity of ASEP, paired with the observed overlap between diet groups, suggests the method could compliment and elaborate on other research methods to measure ASF use.

When calculating ASEP for the EAT-Lancet commission’s planetary health reference diet^5^, we found that the reference diet averages to ASEP of approximately 14%. This predominantly plant-based diet is designed to meet the nutritional requirements for healthy adults and children over two years of age, while also promoting environmentally sustainable food systems. Even though different ASF subgroups have distinct environmental impacts, the share of ASF consumption collectively is the main determinant of the greenhouse gas footprint in European dietary patterns^17^. While ASEP as such is not included in the EAT-Lancet’s set of targets, it is noteworthy that the ASEP calculated for the reference diet equals less than half of the observed median ASEP of omnivorous diet in our study. Thus, ASEP could offer a simple and approachable way to communicate the need to reduce global ASF consumption to promote planetary health.

On group- and individual-level dietary intakes, ASEP demonstrated promising properties. We approximated the required days of food record to estimate ASEP of a non-vegan diet with two slightly different methods. For group-level studies, four days of food record provided sufficient (as defined in^18^) data for correlation analyses, and for individual assessment, 11 days of food record allows an ASEP estimate that has 95% probability to be within +/-5% units from the real ASEP. These results compare well to previous estimates of required food record days in Western and Asian populations, both adult and children, to assess energy intake^19–21^.

One child in our study obtained an ASEP of 0% despite reporting occasional animal food consumption in the dietary screener. Dietary habits maintained over time likely have a greater impact on chronic diseases than occasional departures from a regular diet. In specific scenarios, a more thorough assessment based on longer recording period might be necessary. Thus, depending on the research questions, a food frequency questionnaire or a dietary screener covering e.g., the month prior to sample collection of the study may add essential detail to ASEP calculated from food records.

Correlation analyses showed significant associations between ASEP and several nutrient intakes. In this study, a diet increasingly emphasizing animal foods was associated with higher SAFA and lower PUFA intakes, higher dietary cholesterol intake, and lower fibre and folate intake. There was a positive association of protein intake and ASEP, but none of the participants fell below the recommended protein intake for children over 2-years in Finland (10–20 E%)^22^. These findings are generally in agreement with a recently published systematic review, which compared nutrient intake and nutritional status indicators of children and adolescents on vegetarian or mixed diet^23^.

Strong correlation of ASEP and fatty acid intakes could be especially important for cardiovascular health, as isocaloric replacement of SAFA with PUFA has a substantial LDL-C lowering effect^24^. The noted correlations of ASEP with nutrient intakes are related to the types of food subgroups consumed. Globally, small children consume a higher proportion of their ASF as milk and have less varied sources of ASF compared to adolescents^16^. Previously published results from MIRA Helsinki Study show that the two largest ASF groups contributing to total energy intake among omnivores were “Milk and dairy products” (21E%) and “Meat and meat dishes” (12E%)^25^, also identified to be key contributors of SAFA intake for Finnish children attending daycare^26^. Additionally, fish consumption was low, with “Fish and fish dishes” together contributing to 3E% and 2E% among omnivores and vegetarians, respectively^25^. These food subgroup characteristics are likely reflected in the observed correlations of ASEP with SAFA and PUFA.

One of the strongest associations between ASEP and nutrient intake was observed with dietary fibre. A negative correlation was expected, as fibre is not naturally present in ASF. Almost all study participants met the lower threshold for dietary fibre intake set for children over two years of age in the Finnish national dietary recommendations (2–3 g/MJ)^22^. In fact, most children had fibre intake above this range, with intakes twice as high (4–6 g/MJ) not uncommon. Even though increased fiber intake in adults is associated to health benefits, such as lower cardiometabolic risk, consequences of high fibre intakes in children remain poorly understood^27^. In general, our findings on high fibre intake in children consuming more plant-based diets are consistent with previous studies^27^.

We observed a strong positive correlation between ASEP and LDL-C in the context of the Finnish food environment with generally very high ASF intake. Dietary manipulations generally seem to cause only modest (from 4 to 13%) reductions in cholesterol levels^28^. Besides the magnitude of such reduction, its duration is of importance. Reductions in childhood could be more meaningful as the cumulative risk burden accumulates over life course^12^. Interestingly, certain types of vegan diets, such as the portfolio diet, have in clinical trials been shown to be effective in improving blood lipid concentrations, leading to a cholesterol lowering effect similar than those induced by a typical starting dose of statin treatment^28,29^. It is worth noting that the portfolio diet is not only plant-exclusive, but also specifically emphasizes on cholesterol-lowering foods^29^. However, to verify the association between ASEP and LDL-cholesterol noted in this study, larger future studies with adjustment for confounding are required.

The way ASEP is linked to dietary characteristics depends on individual food subgroup choices, which are modified by various cultural, social, and economical factors. Therefore, the findings are likely to differ among populations and our results should be interpreted accordingly. It is also necessary to emphasize that children have exceptional nutritional needs for growth and development^9^, and nutrient adequacy of diets low in ASF requires special attention. Among MIRA Helsinki study participants, nutrient intakes were generally adequate with most concerns relating to vitamin D, vitamin A, essential amino acid and DHA status, as described in more detail in a previous publication^25^.

Our untargeted metabolomics data revealed relatively understudied IAA to correlate substantially with ASEP. Serum levels of IAA were found to be higher in diets that are more plant-predominant. This is noteworthy because most established dietary biomarkers either accumulate more in omnivorous diets or are suggested to be specific to the intake of certain animal derived foods^30^. The results are consistent with the study of comparative MS metabolomics between adult vegans and omnivores^31^, where indoleacrylate was among the top 30 important separating metabolites in a random forest -based analysis. IAA is a plant growth hormone that cannot be synthesized by animal cells, and is thought to enter human circulation as a metabolic product of intestinal microbiota^15,32^. Thus, high IAA level in serum may directly indicate intake of plant-based foods or reflect the accommodation of intestinal microbiota to more plant-based diet. The knowledge on the effects of IAA for human metabolism and health is scarce, but on molecular level it reduces the activity of tryptophan synthetase, an enzyme required for tryptophan synthesis from indole compounds, which is generally absent in animal cells^33^. As tryptophan is known to be a major regulator of human gut-brain axis^34^, altered levels of IAA in intestinal lumen could modulate gut-brain signaling through this mechanism. As potential confounding factors for the biomarker role of IAA, recent studies have proposed that serum IAA levels increase after fecal transplantation in patients with slow transit constipation^35^ and decrease in various neurological disorders^36^ and diabetic kidney disease^37^. Overall, in this exploratory analysis serum IAA emerged as a potential candidate for more plant-based diets, and as increased IAA levels correlate substantially with dietary choices, its metabolic effects require further studies.

Pipecolate, a metabolite of alternative lysine degradation pathway in human cells and intestinal bacteria^38,39^, showed a strong negative correlation with ASEP. This is contrary to previously reported lower serum lysine concentration and lysine intake in adults consuming vegan and other plant-predominant diets than in those with more animal-predominant diets^40,41^. Indeed, lysine is recognized as the first limiting amino acid in many cereal protein sources such as oats, rice, and wheat^42^. However, high amounts of pipecolate in beans makes it a recognized metabolic marker of bean consumption^43^, partly explaining the discrepancy between pipecolate and lysine levels in plant-forward diet consumers. Our results on IAA and pipecolate indicate that the contribution of the intestinal microbiome should be considered when assessing the availability of lysine in plant-predominant diets. Further studies on metabolic biomarkers of ASEP should include targeted measurements of IAA and other potential biomarkers to ensure accurate identification of the molecules, as well as experimental designs to allow investigation of causal relationships.

Most of the top positively correlating metabolites with ASEP were glycerophospholipids or metabolites highly correlating with glycerophospholipids such as aralia cerebroside (see Supplementary Fig. 2). Methylhistidine, an established meat intake biomarker^44^, and (Z)-4-heptenal, one of the possible volatiles responsible for fishy odors in fish oil^45^, formed additional more independent molecular clusters with higher serum levels seen with animal-predominant diets. Relevance of the most strongly positively correlating metabolite aralia cerebroside for human metabolism is not clear as it has been relatively scarcely studied^46^. However, as aralia cerebroside and glycerophospholipids show high intercorrelation and correlation with LDL-C, and glycerophospholipids are known to mostly travel in serum on the lipid monolayer of lipoprotein particles^47^, the observed statistical associations may be derivative from the strong correlation of plasma LDL-C with ASEP in our dataset. On the other hand, the only lysophospholipid on Supplementary Table 2 showed a strong negative correlation. Lysophospholipids are produced mostly from phospholipids by phospholipase A-type (PLA) enzymes or by lecithin-cholesterol acyl transferase (LCAT)^48^. Further investigation of all phospholipids and lysophospholipids in our dataset (Supplementary Table 3) revealed that the pattern of significant positive correlations of ASEP with phospholipids and negative correlations with lysophospholipids extend to the whole (lyso)phospholipidome detected by MS. Further studies with targeted methods are required to answer whether the suspected increase in lysophospholipid to phospholipid ratio in subjects following plant-based diets has clinically relevant effects in human health.

Strengths of our study are the careful collection and recording of food record data and a sample representing a wide spectrum of ASF consumption. The precision of collected food record data enabled detailed partitioning of consumed foods into ingredients. When approximating animal ingredient proportion of mixed origin food items, we were not able to account for differences in energy yield per mass unit that varies among ingredients with different macronutrient composition. However, of the 719 food items in our food records, only 63 (9%) food items contained both plant- and animal source ingredients and required more subjective assessment. Among the non-vegan participants, the food items with both plant- and animal source ingredients collectively accounted for less than 10% of the estimated total energy intake. Of these food items, one third (*n* = 21) were margarines or fat spreads with fat as the only major energy-yielding macronutrient.

Some database related issues, commonly encountered in food record data collection, complicated the ASEP scoring in general. A small number of food items had been recorded using the food composition database’s generic item codes (e.g., “Cooking fat, average”). For these generic food codes, we estimated the weight proportion of animal source ingredients based on ingredient composition and nutrient profile of similar products. Despite our efforts for meticulous data collection, food records and dietary screeners are always subject to recall bias which may cause measurement error. In addition, reliance on food record data may be seen as a limitation of the ASEP method as data collection may be impractical in large-scale studies.

The use of categorical definition of the child’s daycare diet for recruitment and the homogeneous background of participants may limit the generalizability of the results, in addition to the limited sample size which was mostly impacted by the generally small number of children consuming a vegan diet in daycare even in the largest city of Finland at the time of recruitment. The same background characteristics associated with vegetarianism in adults, such as healthy lifestyle habits^49^, are likely to affect children in families choosing a vegan daycare diet.

## Conclusions

Based on our findings, calculation of ASEP from food records offers a feasible method to study characteristics of dietary patterns. Future studies in different populations and food environments are needed to understand how food subgroup choices influence the association of ASEP and nutrient intakes. IAA among other promising metabolic biomarkers of ASEP should be further studied with targeted methods and larger samples. This could enable the development of objective measures of dietary pattern from biological samples, perhaps utilizing a combination of selected biomarkers with little intercorrelation. We suggest that continuous approaches to measure dietary pattern bring additional value to the research of plant-based diets.

## Methods

### Participants

We used the data from a cross-sectional MIRA Helsinki Study collected from children in early childhood education and care centers (hereinafter referred to as daycare) in Helsinki in 2017. The recruitment and data collection processes have been described in detail elsewhere^25^. The MIRA Helsinki Study was set out to compare the nutrient intake and nutritional statuses of children who had either chosen a vegan meal option or had made no special meal choice in a municipal daycare in Helsinki. The present study included 51 children, out of whom 20 consumed vegan meals in the daycare and followed either a vegan, vegetarian (including pesco-vegetarians) or omnivore diet at home, and 31 children, who consumed omnivorous meals in both daycare and at home. The participants were further classified by their actual diet as described below in “Diet categorization”.

This study was approved by the coordinating ethics committee of Helsinki University Hospital and was performed in line with the principles of the Declaration of Helsinki. At least one caregiver of each child provided a written informed consent.

### Food records and blood samples

The participating families completed a 4-day food record consisting of three weekdays and one weekend day. Food record days of poor food intake due to illness or missing data points were omitted. We used 47 four-day and four three-day food records (total 51 food records) to validate the number of days required for ASEP evaluation. To assist portion size estimation, we provided a validated portion size guide booklet^50^. Dietary intakes were computed with AivoDiet-dietary software (version 2.2.0.1, Aivo Oy, Finland), using the Finnish National Food Composition Database (Fineli) Release 16 (2013), maintained by the Finnish Institute for Health and Welfare. We collected detailed information on products and recipes from the city of Helsinki’s daycare food services. We used the information provided by the food producers to fill in and update nutritional information of the products missing from the database, with new recipes added when necessary. In addition, we had information from caregiver-filled screener covering the child’s food consumption frequency of 68 food groups during the previous week.

A fasting venous blood sample collected after overnight fast was available from 40 of the 51 (78%) participants. For evaluating the association of ASEP with selected blood biomarkers and serum untargeted metabolomics, we calculated ASEP using all available (three (*n* = 3) or four days (*n* = 37)) food record data for each participant with a blood sample. Plasma LDL-C and erythrocyte folate levels were selected as exemplar clinical biomarkers typically corresponding to either extreme of ASF consumption^23,51,52^. These targeted analyses were conducted in a clinically accredited HUSLAB laboratory after sample collection, and a further aliquot was used for untargeted metabolomics after storage at -80 °C as described below.

### Diet categorization

We used combined food record and dietary screener data to classify the children (*n* = 51) into diet categories based on their overall food consumption. For this study, omnivore, vegetarian (criterion: no red meat or poultry) and vegan (criterion: no animal sourced products) diet categories were used. Majority (65%) of the children consuming a vegan diet at daycare consumed some ASF outside daycare. From the children consuming vegan food at daycare (*n* = 20), seven were classified to a vegan, 11 to a vegetarian, and two to an omnivorous diet group. All children consuming omnivorous diets at daycare (*n* = 31) were classified as omnivores.

### Food item scoring and calculation of animal source energy percentage

To examine dietary patterns with different proportions of ASF, we developed a quantitative continuous variable describing the relative average intake of animal source energy. ASF included all foods and drinks made directly of animals (meat, poultry, fish, seafood) or derived from animals, such as eggs, dairy and honey. ASEP was computed from the food record data in two phases: (1) scoring of food items based on their animal source weight proportion and (2) calculating participant’s intake of animal source energy as a percentage of total energy intake across the food record period.

For food item scoring, whenever possible the composite dishes were disaggregated into ingredients. All animal-origin ingredients were given a score 1 and those of plant-origin a score 0. Some foods and drinks had been entered into the database as products/composite dishes, which could not be broken down to ingredients. For such products with both animal and plant source ingredients, we applied the following principles. All dairy products (yogurts, cheeses, ice-creams) were assigned a score of 1. Some of these products contained small amounts of plant-origin ingredients such as fruit, berries, or sugar. However, the weight proportion of such non-animal ingredients was estimated to be small (< 10%). Similarly, sausages and cold cuts containing meat were assigned a score of 1. We estimated that the energy-yielding ingredients in these products came almost exclusively (> 90%) from animal sources. Supplementary Fig. 3 depicts the process of food item scoring, with examples on food products.

The rest of the products were food items with both animal- and plant-origin ingredients. For these items, the proportion of animal-origin ingredients was either calculated or estimated by a researcher. Information on the food products was obtained from the Finnish Food Composition Database Fineli^53^, which has marked products with zero animal content with a label ‘vegan’. In addition, we used websites of the largest Finnish grocery retail trade groups (S-ryhmä, Kesko and Lidl) as well as food manufacturers’ websites to search information on the ingredients. When the exact ingredient proportions were not available, we calculated the relative weight content of animal ingredients from an online recipe of a similar product, or estimated it based on the recipes available on comparable products.

When estimating the proportion of animal ingredients, we did not consider added water, as it does not contribute to energy intake. For the same reason, ingredients present in very small amounts (such as food additives, which in other circumstances could classify an item non-vegan) were not considered. A list of all consumed food items together with the calculated animal proportions is openly available in GitHub (see Data Availability section).

After food item scoring, ASEP was calculated for each participant across the food record period as follows:

$$ASEP = \frac{{\mathop \sum \nolimits_{i}^{~} \left( {E_{i} \cdot ASWP_{i} } \right)}}{{\mathop \sum \nolimits_{i}^{~} E_{i} }},$$

where *I* denotes each food item, *E*_*i*_ energy intake derived from food item *i* and *ASWP*_*i*_ the estimated weight proportion of animal ingredients in food item *i*. Thus, ASEP may obtain values from 0 to 1 (0–100%).

### ASEP in the EAT-Lancet planetary health reference diet

To compare our results to an established dietary pattern, we calculated an estimate of ASEP within the EAT-Lancet Commission’s planetary health diet^5^. The computation was based on given energy intakes (kcal/day) from different food groups for a total intake of 2500 kcal/d. From the food groups listed, we defined “Whole milk or derivative equivalents”, “Beef and lamb”, “Pork”, “Chicken and other poultry”, “Eggs”, “Fish” and “Lard or tallow” as 100% animal source foods, and everything else as 100% plant source foods. Even though in the reference diet the intake of “Chicken and other poultry” is indicated to be exchangeable with plant protein sources, and the intake of “Lard or tallow” is marked as optional, these were included in our calculation as foods contributing to animal source energy only.

### Variability of ASEP

To evaluate the number of food record days required for estimating *ASEP* at reasonable accuracy, we adapted two methods. Beaton’s method^54^ is originally used to calculate food record days required to have a maximum of *p* percentage deviation of the individual’s estimated intake from the real unknown intake of a nutrient with 95% confidence:

$$D_{{Beaton}} = \left( {\frac{{1.96}}{p}CV_{w} } \right)^{2},$$

where *CV*_*w*_ is the intraindividual coefficient of variation, sometimes referred to as “within-subject coefficient of variation”. However, when studying individuals with low ASEP it would be often reasonable to accept higher percentage, but not absolute deviation from real ASEP than with individuals with high ASEP. Thus, we evaluated it is reasonable to estimate food record days required for maximal amount of absolute deviation $\:a=p\cdot\:{m}_{ASEP}$, where $\:{m}_{ASEP}$ is the mean ASEP for the studied population. With the definition of $\:C{V}_{w}=\frac{{s}_{ASEP}}{{m}_{ASEP}}$ we may then substitute:

$$D_{{Beaton}} = \left( {\frac{{1.96 \cdot m_{{ASEP}} }}{a}CV_{w} } \right)^{2} = \left( {\frac{{1.96 \cdot m_{{ASEP}} \cdot s_{{ASEP}} }}{{a \cdot m_{{ASEP}} }}} \right)^{2} = \left( {\frac{{1.96}}{a} \cdot s_{{ASEP}} } \right)^{2} .$$

where $\:{s}_{ASEP}$ is the intraindividual standard deviation of daily ASEP that we estimate with $\:\widehat{{s}_{ASEP}}$, weighted mean of standard deviations calculated for each individual:

$$\widehat{{s_{{ASEP}} }} = \frac{{\mathop \sum \nolimits_{i}^{~} d_{i} \cdot s_{{ASEP,i}} ~}}{{\mathop \sum \nolimits_{i}^{~} d_{i} }},$$

where

$$s_{{ASEP,i}} = \sqrt {\frac{{\mathop \sum \nolimits_{{day~ = ~1}}^{{d_{i} }} \left( {ASEP_{{day,i}} - m_{{ASEP,i}} } \right)^{2} }}{{d_{i} }}} ,$$

where *m*_*ASEP, i*_ and *s*_*ASEP, i*_ are the mean and standard deviation of recorded daily *ASEP*s of individual *i*, respectively.

Fully vegan diets have a *s*_*ASEP*_ of 0 by definition (*ASEP* for each day would always be 0, thus no deviation from mean occurs), so we assessed the statistical properties of *ASEP* using vegetarian and omnivore participant food records only.

As another approach, we adapted Liu’s method^18^, commonly used for estimating the number of food record days needed for adequate correlation analyses. When estimating true correlation coefficient $\:{\rho\:}_{12}$ between two variables (e.g., ASEP and serum LDL-C) based on measurements from a sample of individuals, the intraindividual variance of both variables causes extra variation in the observed data. This additional variation causes the estimated value R ~ $\:\rho\:$ to shrink towards zero by an error term *e* that will be more significant with variables with higher ratio of intraindividual to interindividual variance, $\:{{\sigma\:}^{2}}_{w}/{{\sigma\:}^{2}}_{b}$, and less observations *n* (e.g. separate measurements per individual, or, in our case, days of food record):

$$R = \rho _{{12}} \cdot e_{1} \cdot e_{2} = \rho _{{12}} \cdot \sqrt {1/\left( {1 + \sigma ^{2} _{{w,1}} /n_{1} \sigma ^{2} _{{b,1}} } \right)} \cdot \sqrt {1/~\left( {1 + \sigma ^{2} _{{w,2}} /n_{2} \sigma ^{2} _{{b,2}} } \right)}$$

As Liu et al. demonstrated, intra- to interindividual variances and, hence, error terms $\:{e}_{i}$ may be estimated in a sufficiently large sample of individuals by calculating the correlation coefficient $\:{r}_{i}$ of two independent measurements (e.g., in our data, daily ASEP’s from two randomly chosen days of an individual):

$$e_{i} \approx \sqrt {1/\left( {1 + \left( {1 - r_{i} } \right)/nr_{i} } \right)}$$

Solving for *n* yields an (in)equation that can be used to determine measurements (i.e. days of food record) needed to achieve an error term greater (more insignificant) than a predetermined error limit. Traditionally, an error term of 0.9 has been considered an adequate limit. Substituting $\:n={D}_{Liu}$ as the notation for “days needed”:

$$D_{{Liu}} = \frac{{e_{i} ^{2} }}{{1 - e_{i} ^{2} }} \cdot \frac{{1 - r_{i} }}{{r_{i} }} = \frac{{0.9^{2} }}{{1 - 0.9^{2} }} \cdot \frac{{1 - r_{i} }}{{r_{i} }}$$

### Untargeted metabolomics

The untargeted metabolomics data of MIRA Helsinki Study was used to detect possible serum biomarkers for ASEP. The analysis of samples was conducted with flow injection time-of-flight (TOF)-MS on an Agilent 6550 QTOF instrument in negative mode exactly as described by Fuhrer et al.^55^. Putative annotations were created based on Human Metabolome Database v.3.6 using both accurate mass per charge (tolerance 0.001 *m*/*z*) and isotopic correlation patterns, allowing the detection of 872 metabolites. The approach is sufficient to assign molecular formulas in most cases, but not to distinguish between isomers. A more detailed description of data acquisition and a comprehensive list of detected metabolites has been described in a previous article^25^.

### Statistical analyses

Statistical analyses were implemented in Python (version 3.11.5) and R (version 4.2.1) programming languages using open-source statistical software libraries. Source code is openly available (see Data availability section). Associations of ASEP with dietary intakes, and plasma LDL-C and erythrocyte folate concentrations were studied using Spearman correlation coefficient. The level of statistical significance was assigned based on significant (non-parametric Spearman) correlation, assuming Student’s t-test for significance of correlation and Bonferroni multiple testing correction separately for correlation analyses of 12 intake and clinical blood biomarker values, and 872 untargeted metabolomics values. Threshold for significant correlation was chosen to correspond to Bonferroni corrected *p* < 0.05. This yields significant (Spearman) correlation coefficient of |r| > 0.40 for targeted intake and clinical blood biomarker data and |r| > 0.54 for untargeted metabolomics data. For untargeted metabolomics data, Spearman correlation coefficient to ASEP was calculated for each metabolite and as we assessed the sample size allows exploratory analyses at most, correlation coefficient was considered as more relevant proxy for connection with ASEP than p-value from a statistical test of any type. The aforementioned level of significance in correlation coefficient was used merely as a tool to describe patterns in (lyso)phospholipidome. Twenty metabolites with the highest absolute value of correlation coefficient were considered independently as an example set of potential plant-based diet biomarker candidates. Hierarchical clustering was conducted for top 20 metabolites with built-in R-function *hclust*, using complete linkage method and *d = 1 - r*_*Pearson*_ as dissimilarity measure.

## Electronic supplementary material

Below is the link to the electronic supplementary material.

Supplementary Material 1

## Data Availability

Source code for generating figures and data analyses including the food item scoring and a list of all consumed food items and their estimated ASWPs is openly available at: https://github.com/elinakettunen/asestudy. Pandas was used for data analysis and curation. Statistical tests were performed using functions from the statsmodels-module and scipy.stats. Figures were generated with the Seaborn data visualization library (version 0.12.2). Source code for calculations for number of days required in food records is openly available at http://www.github.com/topihovinen/asestudy. The data of this study are not openly available due to risk of identifying single participants and their personal health data. The data are available from the corresponding author upon reasonable request.
