# Supplementary material for: Nutrient intakes and metabolomic profiles associated with animal source energy percentage in children’s diets
Source: Sci Rep. 2025 May 27;15:18568. doi: 10.1038/s41598-025-02114-8 (PMC12116860; doi:10.1038/s41598-025-02114-8)
Supplement: Supplementary file 1 — Supplementary Material 1 [file 41598_2025_2114_MOESM1_ESM.docx]

**Supplementary Information**

**Journal name**: Scientific Reports

**Article name**: Nutrient intakes and metabolomic profiles associate with animal source energy percentage in children's diets

**Authors**: *Topi Hovinen^1^, *Elina Kettunen^2^, Maijaliisa Erkkola^2^, Anu Suomalainen^1,3,4^, Riitta Freese^2^, Liisa Korkalo^2^

*Shared first authorship of Topi Hovinen and Elina Kettunen.

**Affiliations:**

^1^Research Programs Unit, Stem Cells and Metabolism, University of Helsinki, 00290 Helsinki, Finland.

^2^Department of Food and Nutrition, University of Helsinki, 00014 Helsinki, Finland.

^3^HUS Diagnostic Center, Helsinki University Hospital, 00290 Helsinki, Finland.

^4^HiLife, University of Helsinki, 00014 Helsinki, Finland.

**Correspondence** to liisa.korkalo@helsinki.fi

## Supplementary Figure 1


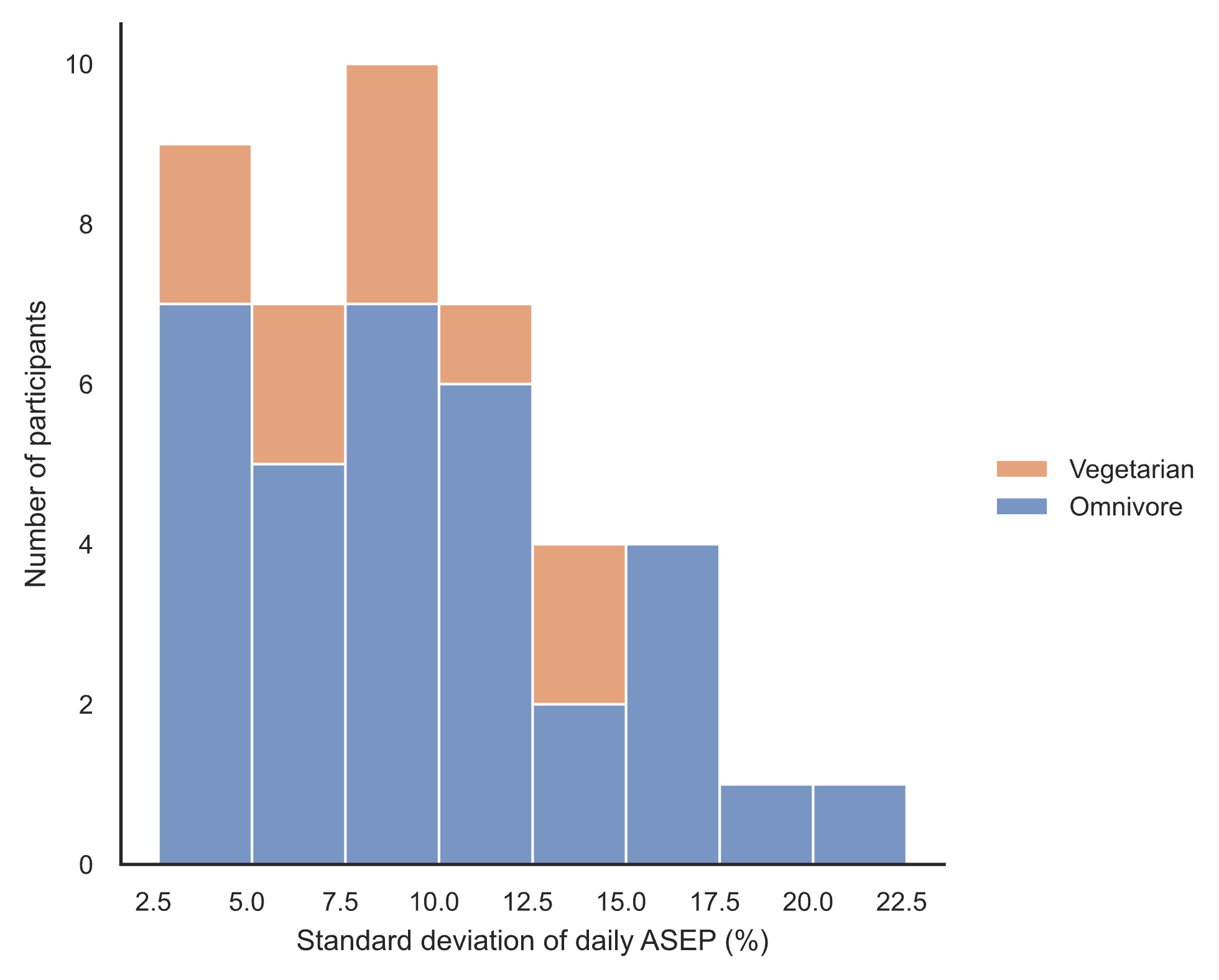


**Supplementary Fig. 1**. The distribution of estimated individual standard deviations of daily animal source energy percentage (ASEP) scores calculated from the 3- or 4-day food records (n=43) of omnivore or vegetarian participants. Vegan diets have an ASEP standard deviation of 0 by definition.

## Supplementary Figure 2


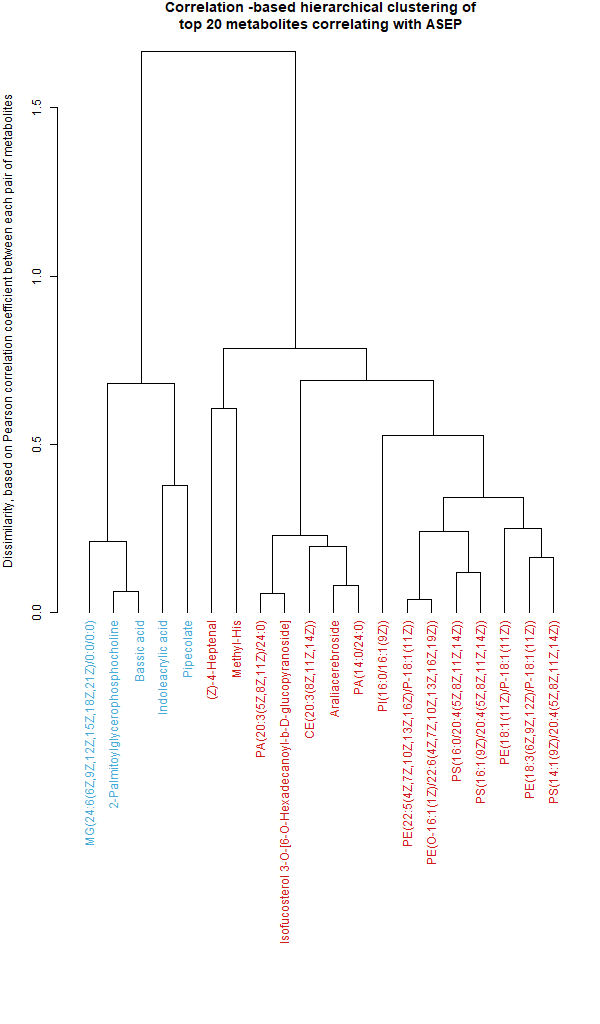


**Supplementary Fig. 2.** Hierarchical clustering of top 20 serum metabolites (from untargeted MS metabolomics) correlating with ASEP. The metabolites are more thoroughly discussed in Supplementary Table 2.

##

## Supplementary Figure 3


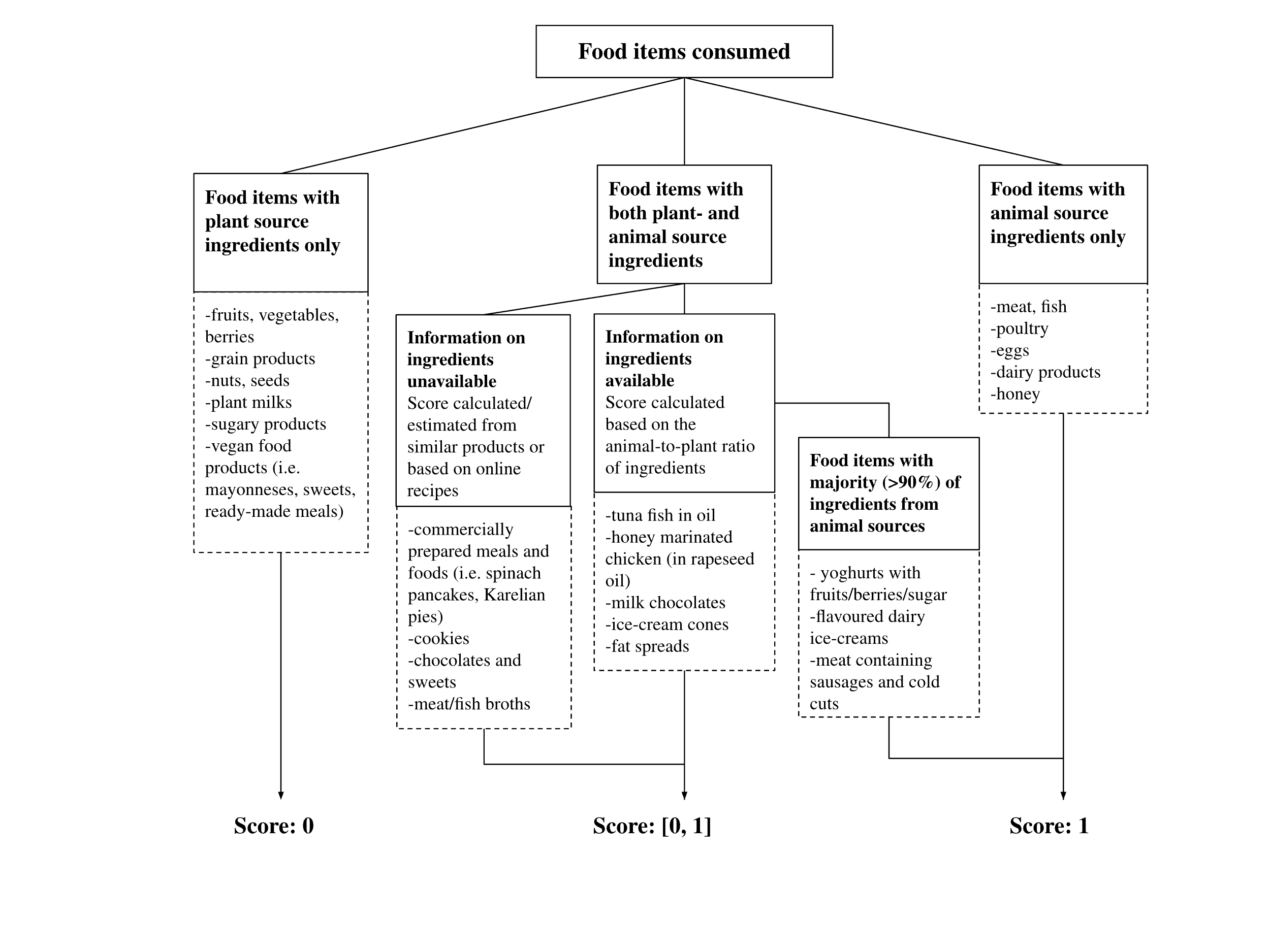


**Supplementary Fig. 3**. The process of food item scoring with examples of food products.

## Supplementary Table 1

**Supplementary Table 1.** Correlation coefficients of ASEP with 10 nutrients and two plasma biomarkers, with Bonferroni correction for these 12 variables.

| **Nutrient intake** | **r_Spearman_** |
| --- | --- |
| Energy (per day) | -0.205 |
| Fat (E%) | 0.094 |
| Saturated fatty acids (E%) | 0.785* |
| Monounsaturated fatty acids (E%) | -0.325 |
| Polyunsaturated fatty acids (E%) | -0.819* |
| Cholesterol (per MJ) | 0.715* |
| Protein (E%) | 0.567* |
| Carbohydrates (E%) | -0.188 |
| Fibre (per MJ) | -0.811* |
| Folate (per MJ) | -0.776* |
| **Biomarker concentration** | **r_Spearman_** |
| Plasma LDL cholesterol (mmol/l) | 0.699* |
| Erythrocyte folate (nmol/l) | -0.521* |

## *Statistically significant correlation: |r| > 0.40, see Methods for reasoning behind statistical significance

## Supplementary Table 2

**Supplementary Table 2.** Top 20 metabolites of highest absolute value of correlation coefficient with animal source energy percentage.

|  | r^1^ | m/z^2^ | Metabolite;  *HMDB ID* | Alternatives^3^ | Description^4^ |
| --- | --- | --- | --- | --- | --- |
| 1 | -0.750 | 186.055608 | Indoleacrylic acid;  *HMDB0000734* | - | Plant growth hormone, metabolite of tryptophan metabolism of intestinal microbiota |
| 2 | 0.741 | 730.5475699 | Aralia cerebroside  *HMDB0033621* | - | A glycosphingolipid recognized in both animal- and plant-based foods |
| 3 | 0.741 | 807.5019 | PI(16:0/16:1(9Z))  *HMDB0009779* | Any phosphatidylinositol with fatty acids totaling 32 carbon atoms and 1 double bond. | Phosphoinositol with palmitic acid and palmitoleic acid as the fatty acid moieties |
| 4 | 0.717 | 731.5582902 | PA(14:0/24:0)  *HMDB0114793* | Any phosphatidic acid with saturated fatty acids totaling 38 carbon atoms., e.g. PA(18:0/20:0) | Phosphatidic acid with myristic acid (14:0) and lignoceric acid (24:0). |
| 5 | 0.713 | 809.605596 | PA(20:3(5Z,8Z,11Z)/24:0)  *HMDB0115142* | Any phosphatidic acid with total of 44 carbon atoms and 3 double bonds | Phosphatidic acid with Mead acid (20:3), lignoceric acid (24:0) and a phosphate moiety occupying glycerol substitution sites. |
| 6 | 0.678 | 782.496309 | PS(16:0/20:4(5Z,8Z,11Z,14Z))  *HMDB0012361* | Any phosphatidylserine with fatty acids of total of 36 carbon atoms and 4 double bonds^5^ | Phosphatidylserine with palmitic acid (16:0), arachidonic acid (20:4) and a phosphorylserine moiety occupying glycerol substitution sites. PS are most abundant in myelin of brain tissue. |
| 7 | 0.667 | 780.481352 | PS(16:1(9Z)/20:4(5Z,8Z,11Z,14Z))  *HMDB0012372* | Any phosphatidylserine with fatty acids of total of 36 carbon atoms and 5 double bonds^5^ | Phosphatidylserine with palmitoleic acid (16:1) and arachidonic acid (20:4) and a phosphorylserine moiety occupying glycerol substitution sites. PS are most abundant in myelin of brain tissue. |
| 8 | 0.667 | 726.541853 | PE(18:1(11Z)/P-18:1(11Z))  *HMDB0009050* | Any phosphatidylethanolamine with one fatty acid and one plasmalogen of total of 36 carbon atoms and 2 double bonds^5^ | Phosphoethanolamine with vaccenic acid, plasmalogen 18:1n7 and a phosphorylethanolamine moiety occupying glycerol substitution sites. |
| 9 | 0.663 | 722.511673 | PE(18:3(6Z,9Z,12Z)/P-18:1(11Z))  *HMDB0009149* | Any phosphatidylethanolamine with one fatty acid and one plasmalogen of total of 18 carbon atoms and 4 double bonds^5^ | Phosphoethanolamine with gamma-linoleic acid, plasmalogen 18:1n7 and a phosphorylethanolamine moiety occupying glycerol substitution sites. |
| 10 | 0.660 | 752.452564 | PS(14:1(9Z)/20:4(5Z,8Z,11Z,14Z))  *HMDB0012350* | Any phosphatidylserine with fatty acids of total of 34 carbon atoms and 5 double bonds^5^ | Phosphatidylserine with myristoleic acid (14:1), arachidonic acid (20:4) and a phosphorylserine moiety occupying glycerol substitution sites. PS are most abundant in myelin of brain tissue. |
| 11 | 0.658 | 774.5418828 | PE(22:5(4Z,7Z,10Z,13Z,16Z)/P-18:1(11Z))  *HMDB0009644* | Any phosphatidylethanolamine with one fatty acid and one plasmalogen of total of 40 carbon atoms and 6 double bonds or 38 carbon atoms and 3 double bonds^5^ | Phosphoethanolamine with docosapentaenoic acid, plasmalogen 18:1n7 and a phosphorylethanolamine moiety occupying glycerol substitution sites. |
| 12 | 0.655 | 673.5951703 | CE(20:3(8Z,11Z,14Z))  *HMDB0006736* | - | Cholesterol ester of eicosatrienoic acid (20:3). |
| 13 | -0.644 | 495.334312 | LysoPC(16:0)  *HMDB0061702* | - | Lysophosphatidylcholine with palmitic acid as the fatty acid moiety |
| 14 | -0.640 | 429.3009996 | MG(24:6(6Z,9Z,12Z,15Z,18Z,21Z)/0:0/0:0)  *HMDB0011590* | **1)** Some mono- (HMDB0012458) or dihydroxy (HMDB0034403) bile acids  **2)** Some triterpenoids (HMDB0030066, HMDB0036249)  **3)** A vitamin D3 derivative (HMDB0060127) | Monoacylglycerol with tetracosahexaenoic acid (24:6) occupying one glycerol substitution site. |
| 15 | 0.637 | 111.081258 | (Z)-4-Heptenal  *HMDB0031483* | **1)** Heptenones (e.g. HMDB0031487)  **2)** Methylcyclohexanones (e.g. HMDB0031540)  **3)** Methyl-hexenones (e.g. HMDB0031550) | (Z)-4-Heptenal has been detected in food products supplemented with fish oil. |
| 16 | -0.634 | 128.0710005 | Pipecolate  *HMDB0000070* | **1)** Mostly plant-derived proline metabolites (e.g. HMDB0094696, HMDB0029435)  **2)** Butanal derivatives (e.g. HMDB0004226, HMDB0059649)  **3)** Cycloleucine (HMDB0062225) | A metabolite in lysine degradation by intestinal bacteria and an abundant component of beans |
| 17 | 0.631 | 746.5109073 | PE(P-16:1(1Z)/22:6(4Z,7Z,10Z,13Z,16Z,19Z))  *HMDB0005780* | Any phosphatidylethanolamine with one fatty acid and one plasmalogen of total of 38 carbon atoms and 7 double bonds or 36 carbon atoms and 3 double bonds^5^ | Phosphoethanolamine with docosahexaenoic acid, plasmalogen 16:1n1 and a phosphorylethanolamine moiety occupying glycerol substitution sites. |
| 18 | 0.629 | 811.6464374 | Isofucosterol 3-O-[6-O-Hexadecanoyl-b-D-glucopyranoside]  *HMDB0032811* | - | A constituent of garden tomato (*Lycopersicon esculentum*) |
| 19 | 0.628 | 168.0772616 | 1-Methylhistidine  *HMDB0000001* | 3-Methylhistidine (HMDB0000479) | Established biomarker of meat [S1] and fish [S2] intake. E.g. urinary 1-MH correlates with red meat and fish intake, 3-MH with white meat intake |
| 20 | -0.626 | 485.3271059 | Bassic acid  *HMDB0034526* | Several other triterpenoids, e.g. glycyrrhetic acid (HMDB0035259), ceanothic acid (HMDB0036851) and actinidic acid (HMDB0037963) derivatives | A triterpenoid found in e.g. fruits. |
| ¹r = Spearman correlation coefficient between metabolite levels and animal source energy percentage (ASEP).  ^2^Mass per charge -value for the ion.  ^3^Mass spectrometer can distinguish metabolites with different m/z values, but often there are multiple known/theoretically possible metabolites with identical m/z value. Suggested annotation shown in “Metabolite” column has the highest predicted probability of being found in human serum sample. Alternative list includes metabolites with identical m/z value that have known role in human metabolism according to Human Metabolome Database (HMDB). However, if the suggested metabolite is the only alternative with known concentrations in human blood, alternative list is not included.  ^4^Information on possible role of the annotated metabolite in metabolism according to HMDB or FooDB unless referenced otherwise.  ^5^Functions and sources of glycerophospholipids vary depending on which fatty acids are included in a specific glycerophospholipid. As the mass spectrometer is unable to distinguish between glycerophospholipids of the same species and number of carbon atoms and double bonds, the correct identification of a possible biomarker in untargeted single MS dataset is challenging. | | | | | |

## Supplementary Table 3

**Supplementary Table 3.** Correlation coefficients of ASEP with all phospholipids (44) and lysophospholipids (17) detected by MS. 13 phospholipids (30%) had a significant positive correlation with ASEP and three lysophospholipids (18%) had a significant negative correlation with ASEP, denoted with white row background and **bold** correlation coefficient. All other metabolites in the list had a negligible correlation with ASEP.

| **LPL** | **PL** | **Metabolite name** | **m/z** | ***r_Spearman_** |
| --- | --- | --- | --- | --- |
|  | X | PI(16:0/16:1(9Z)) | 807.5019 | **0.741** |
|  | X | PA(14:0/24:0) | 731.55829 | **0.717** |
|  | X | PA(20:3(5Z,8Z,11Z)/24:0) | 809.605596 | **0.713** |
|  | X | PS(16:0/20:4(5Z,8Z,11Z,14Z)) | 782.496309 | **0.677** |
|  | X | PS(16:1(9Z)/20:4(5Z,8Z,11Z,14Z)) | 780.481352 | **0.667** |
|  | X | PE(18:1(11Z)/P-18:1(11Z)) | 726.541853 | **0.667** |
|  | X | PE(18:3(6Z,9Z,12Z)/P-18:1(11Z)) | 722.511673 | **0.663** |
|  | X | PS(14:1(9Z)/20:4(5Z,8Z,11Z,14Z)) | 752.452564 | **0.660** |
|  | X | PE(22:5(4Z,7Z,10Z,13Z,16Z)/P-18:1(11Z)) | 774.541883 | **0.658** |
|  | X | PE(O-16:1(1Z)/22:6(4Z,7Z,10Z,13Z,16Z,19Z)) | 746.510907 | **0.631** |
|  | X | PC(18:4(6Z,9Z,12Z,15Z)/P-16:0) | 736.52792 | **0.591** |
|  | X | PA(18:2(9Z,12Z)/24:1(15Z)) | 781.573233 | **0.585** |
|  | X | PA(16:0/22:5(4Z,7Z,10Z,13Z,16Z)) | 721.481817 | **0.556** |
|  | X | PE(22:6(4Z,7Z,10Z,13Z,16Z,19Z)/P-18:1(11Z)) | 772.529517 | 0.519 |
|  | X | PC(18:1(9Z)/18:1(9Z)) | 784.585649 | 0.488 |
|  | X | PA(16:0/18:1(11Z)) | 673.478975 | 0.485 |
|  | X | PA(18:0/24:0) | 787.622327 | 0.472 |
|  | X | PC(15:0/16:1(9Z)) | 716.523414 | 0.446 |
|  | X | PI(18:0/22:6(4Z,7Z,10Z,13Z,16Z,19Z)) | 909.549773 | 0.446 |
|  | X | PE(16:0/22:6(4Z,7Z,10Z,13Z,16Z,19Z)) | 762.506521 | 0.431 |
|  | X | PI(16:0/16:2(9Z,12Z)) | 805.487394 | 0.423 |
|  | X | PC(14:0/20:2(11Z,14Z)) | 756.55395 | 0.407 |
|  | X | PA(22:1(13Z)/19:2(10Z,13Z)) | 767.558947 | 0.402 |
|  | X | PC(14:0/16:0) | 704.522673 | 0.360 |
|  | X | PE(14:0/20:2(11Z,14Z)) | 714.508681 | 0.357 |
|  | X | PC(14:0/15:0) | 690.505595 | 0.348 |
|  | X | PE(22:4(7Z,10Z,13Z,16Z)/P-18:1(11Z)) | 776.558495 | 0.339 |
|  | X | PS(14:0/20:4(5Z,8Z,11Z,14Z)) | 754.465193 | 0.320 |
|  | X | PS(20:2(11Z,14Z)/24:1(15Z)) | 896.639058 | 0.241 |
|  | X | PS(20:3(5Z,8Z,11Z)/22:5(4Z,7Z,10Z,13Z,16Z)) | 858.52977 | 0.238 |
|  | X | PE(20:0/24:1(15Z)) | 856.680629 | 0.226 |
|  | X | PA(16:0/22:6(4Z,7Z,10Z,13Z,16Z,19Z)) | 719.46412 | 0.214 |
|  | X | PS(18:0/24:0) | 874.655934 | 0.207 |
|  | X | PS(DiMe(11,3)/MonoMe(11,3)) | 850.486479 | 0.192 |
| X |  | LysoPE(0:0/16:0) | 452.277419 | 0.190 |
| X |  | LysoPC(20:3(5Z,8Z,11Z)) | 544.342173 | 0.183 |
|  | X | PA(16:0/18:2(9Z,12Z)) | 671.464051 | 0.154 |
|  | X | PC(18:0/24:1(15Z)) | 870.697278 | 0.151 |
|  | X | PC(15:0/20:4(5Z,8Z,11Z,14Z)) | 766.536985 | 0.137 |
|  | X | PC(15:0/18:2(9Z,12Z)) | 742.538187 | 0.104 |
|  | X | PS(DiMe(11,3)/MonoMe(11,5)) | 878.517482 | 0.100 |
|  | X | PS(DiMe(11,3)/MonoMe(13,5)) | 906.548451 | 0.098 |
| X |  | LysoPA(0:0/18:1(9Z)) | 435.251042 | 0.073 |
|  | X | PS(DiMe(13,5)/DiMe(13,5)) | 976.627389 | 0.057 |
| X |  | LysoPA(a-13:0/0:0) | 367.188093 | 0.046 |
| X |  | LysoPC(15:0) | 480.30871 | 0.040 |
| X |  | LysoPE(0:0/22:6(4Z,7Z,10Z,13Z,16Z,19Z)) | 524.276438 | 0.040 |
| X |  | LysoPE(0:0/20:1(11Z)) | 506.323628 | 0.008 |
|  | X | PA(8:0/16:0) | 535.339804 | -0.028 |
| X |  | LysoPE(0:0/18:1(11Z)) | 478.291966 | -0.051 |
| X |  | LysoPA(0:0/18:2(9Z,12Z)) | 433.234374 | -0.058 |
|  | X | PA(18:4(6Z,9Z,12Z,15Z)/18:4(6Z,9Z,12Z,15Z)) | 687.40199 | -0.086 |
| X |  | LysoPI(0:0/20:4(5Z,8Z,11Z,14Z)) | 619.289404 | -0.185 |
|  | X | PA(8:0/18:0) | 563.369719 | -0.192 |
| X |  | LysoPA(0:0/20:4(5Z,8Z,11Z,14Z)) | 455.219297 | -0.225 |
| X |  | LysoPE(0:0/20:2(11Z,14Z)) | 504.308636 | -0.248 |
| X |  | LysoPE(0:0/20:4(5Z,8Z,11Z,14Z)) | 500.277612 | -0.321 |
| X |  | LysoPC(10:0) | 411.24034 | -0.352 |
| X |  | LysoPC(20:0) | 550.389631 | **-0.569** |
| X |  | LysoPC(18:1/0:0) | 521.350375 | **-0.590** |
| X |  | LysoPC(0:0/16:0) | 495.334312 | **-0.644** |
| *Statistically significant correlation: \|r\| > 0.54, see Methods for reasoning behind statistical significance | | | | |

## Supplementary References

1. Said, M. Y. *et al*. Meat intake and risk of mortality and graft failure in kidney transplant recipients. *Am. J. Clin. Nutr.* **114(4)**, 1505–1517. <https://doi.org/10.1093/ajcn/nqab185> (2021).
2. Solvik, B. S. *et al*. Biomarkers and Fatty Fish Intake: A Randomized Controlled Trial in Norwegian Preschool Children. *J. Nutr.* **151(8)**, 2134–2141. <https://doi.org/10.1093/jn/nxab112> (2021).
